# Reply to Marmor: Multiple perspectives for appreciating the meaning and beauty of Turner and Monet paintings

**DOI:** 10.1073/pnas.2303372120

**Published:** 2023-04-10

**Authors:** Anna Lea Albright, Peter Huybers

**Affiliations:** ^a^Laboratory of Dynamic Meteorology, Sorbonne University, École normale supérieure, 75005 Paris, France; ^b^Department of Earth and Planetary Sciences, Harvard University, Cambridge, MA 02138

Marmor agrees with us and others that, “[b]oth Turner and Monet documented London haze when they encountered it” but argues that “[t]he body of their work evolved over a lifetime to emphasize color, light, and impressions for personal and philosophical reasons unrelated to London pollution” (1). The way our hypothesis could be wrong is if changes in air pollution did not influence Turner and Monet’s paintings, but we find this counterfactual difficult to reconcile with various art historical, theoretical, and empirical lines of evidence.

There is precedent for interpreting Turner and Monet’s works in the context of changes in atmospheric properties. Turner accurately rendered sunset coloration as expected following a volcanic eruption (2). Industrialization led to substantial increases in pollution over their careers (3), and art historical literature discusses a relationship between air pollution and their painting style (4–7). In our manuscript, we present theoretical, empirical, and further contextual evidence for this hypothesis (8). Monet himself wrote he was “terrified to see that there was no fog … but gradually the fires were lit and the smoke and haze came back” (9), and “What a sad day this English Sunday … no train, no smoke, no boat, nothing to excite the inspiration” (10).

Marmor states he would have no quarrel with our study if the title, “Paintings by Turner and Monet depict trends in 19th century air pollution,” was prefaced by the word, “Selected.” Marmor’s title would be more precise, but we made no claim to describing all of Turner or Monet’s works. Rather, we chose paintings in and around London and Paris spanning the development of their impressionistic work.

With respect to referencing eye disease, we acknowledge our mistake in citing Marmor’s work as indicating that Degas suffered from cataracts instead of progressive retinal disease. As we do not analyze Degas paintings, this mistake had no further implications. As for our “referencing an erroneous and long-outdated 19th century article to consider eye disease in art,” this was merely to show the issue has long been considered. More generally, it seems incongruous for Marmor to argue that the physiology of seeing can be important for how art is rendered (11) but properties of the atmosphere through which light passes are immaterial.

Let us close by emphasizing that our findings relate to select features of specific works of art. Trends in the blurriness and palette across 116 paintings accord with the expected effects of increasing atmospheric pollution, even after controlling for other factors. This correspondence suggests that Monet, Turner, and others drew inspiration from their environment, but we hasten to add that such a connection is not exclusive of other influences. Ophthalmology, atmospheric sciences, and an array of other specialized disciplines may each have something to offer for appreciating the meaning and beauty of certain paintings. Finally, if there is a connection between Impressionism and the physical environment, it does not detract from the artists’ agency—rather, bringing forth what is real, but not before entirely seen, seems powerfully creative.
